# Adherence to self-care recommendations and associated factors among adult heart failure patients. From the patients’ point of view

**DOI:** 10.1371/journal.pone.0211768

**Published:** 2019-02-07

**Authors:** Mohammed Assen Seid, Ousman Abubeker Abdela, Ejigu Gebeye Zeleke

**Affiliations:** 1 Department of Clinical Pharmacy, University of Gondar, Gondar, Ethiopia; 2 Department of Epidemiology and Biostatistics, Institute of Public Health, University of Gondar, Gondar, Ethiopia; Qazvin University of Medical Sciences, ISLAMIC REPUBLIC OF IRAN

## Abstract

**Background:**

Nowadays, heart failure (HF) related morbidity and mortality rate is increasing globally. Younger populations happen to be more affected by HF in sub- Saharan African than the western countries. Even though medications, low sodium diet, regular exercise, and weight monitoring are essential to control heart failure symptoms and its exacerbation, poor adherence to these self-care recommendations is contributing to an increased in hospitalization, morbidity, and mortality. Therefore, this study aimed to assess heart failure patients’ adherence to self-care recommendations and its associated factors.

**Methods:**

A hospital-based cross-sectional study was conducted on 310 adult heart failure patients attending Gondar University referral hospital from February to May 2017. The participants were selected by systematic random sampling technique. Data were collected through face to face interview and from the patients’ medical records. The data were analyzed using SPSS version 20. A binary logistic regression model was used to check the effect of different factors on the patients’ adherence level.

**Results:**

Of 310 study participants only 22.3% (95% CI, 17.4%-26.8%) of heart failure patients reported good adherence to their self-care recommendations. Adherence to self-care recommendation was positively associated with being male in gender (AOR = 2.34, 95% CI: 1.18–4.62), good level of heart failure knowledge (AOR = 2.49, 95% CI: 1.276–4.856) and free from chronic comorbid diseases (AOR = 2.57, 95% CI: 1.28–5.14).

**Conclusion:**

Overall, heart failure patients’ adherence to self-care recommendations is poor and selective. Being male in gender, had no chronic comorbidity, and a good level of heart failure knowledge were positively associated with adherence to self-care recommendations. It is therefore strategic to plan improving heart failure patients’ knowledge about heart failure signs, symptoms and its management approaches, to improve the patients’ adherence level.

## Introduction

Heart failure (HF) is an ultimate clinical outcome, resulted from either structural or functional dysfunction of ventricular filling or ejection of blood” [1]. HF is a rapidly growing cardiovascular disorder which affects more than 37.7 million individuals worldwide [2].

In high economic countries like the USA, heart failure has affected over six million adult populations (≥20 years of age) [3]. On the basis of the 2013 death rate report, more than 2200 patients were died due to cardiovascular disease each day. Heart failure had been the underlying cause for the majority of those deaths [4].

Currently, heart failure is also omnipresent throughout sub-Saharan Africa; which covers around 44% of newly diagnosed cardiovascular diseases [5]. From the sociodemographic perspective, younger populations happen to be more affected by HF in sub -Saharan African than the western countries [6,7]. The relatively early onset of HF in low economic regions is explained by the frequent recurrence of rheumatic fever, rheumatic valvular heart diseases, congenital heart disease, and infective endocarditis. These bacterial infections are precursors for the development of acute heart failure [7,8]. Currently, the rate of heart failure associated deaths is higher in middle and low-income countries than in high-income countries [9].

The onerousness of HF is further extended to encompass the overall prognosis of heart failure patients, resulting in significant functional impairment, and symptom burden [4,9]. The contributing factors for the poor prognosis of HF include the presence of co-morbidities, the severity of the disease, and inadequate health literacy. Furthermore, HF patients age variation and gender reported to have an impact on HF progression [6,10–12]. Studies have also shown unfavorable HF patients prognosis attributable to poor adherence to self-care recommendations [13–15].

Treatment for heart failure typically consists of a complex regimen of medications, low sodium diet, regular exercise, and weight monitoring. The amalgamation of these regimens is essential to enhance the patients’ functional capacity, improve quality of life, prevent hospital admission, and reduce mortality and morbidity [16,17]. However, poor adherence to these treatment approaches continues to be an extensive problem for HF patients and their family [18].

As elucidated by World Health Organization (WHO), adherence to long-term therapy is “the extent to which a person’s behavior–taking medication, following a diet, and/or executing lifestyle changes, corresponds with agreed recommendations from a health care provider”[19]. Self-care in heart failure is a “naturalistic decision making process”[20] that consists of implementing a number of HF-related practices such as, taking low sodium diet, taking prescribed medications appropriately, keeping physically active, monitoring for symptoms of fluid retention by body weight measurement, and limiting excess fluid intake [21].

As previous studies indicated that most HF patients take less than two-thirds of their prescribed medications[15] and exhibit a polarizing adherence rate in sodium diet restriction (28% to 88%) [22–29], body weight monitoring (2.5% to 83%) [22–26,30,31]. These low adherence is associated with higher incidence of hospitalization, mortality, and morbidity [14,15,31]. Some studies in Africa reported that adherence to self-care recommendations varied from 2.5 to 98% [26,27]. Mostly, HF patients had low adherence to high sodium diet restriction, regular exercise, weight monitoring, and fluid intake restriction in Africa including Ethiopia [22,27].

Despite the increasing trend of HF related morbidity and mortality in Africa, including Ethiopia [32,33], there is scant of evidence regarding heart failure patients adherence to self-care behaviors. Therefore, this study was intended to assess heart failure patients’ adherence to self-care recommendations and to identify factors which would affect the patients’ adherence level.

## Methods and materials

A hospital-based cross-sectional study was conducted at Gondar University referral hospital from February to May 2017. The hospital is located in Gondar town, Northwest Ethiopia, 738 kilometers away from Addis Ababa, the capital city of Ethiopia. The hospital has a wide range of health care services for the residents. There are four main departments (internal medicine, pediatrics, surgery, and gynecology) holding the lion–share in health care delivery. The department of internal medicine dedicates once per week follow up service for HF patients in the outpatient department. Based on the patient’s clinical condition and place of residence (rural or urban) the appointment for follow-up is either monthly or every three months.

Patients who met the inclusion criteria were incorporated in the study: patients who were 18 years old or above, had been diagnosed with HF, and taking medications and had at least a one month follow up prior to the commencement of the study. A total of 310 heart failure patients have met the inclusion criteria and included in this study.

Ethical clearance was obtained from the ethical review committee of the University of Gondar. Permission letter also obtained from the department of clinical pharmacy and chronic outpatient department (OPD) coordinator. Participants were informed about the purpose of the study, full right to discontinue or refuse to participate. Verbal consent was obtained from each participant before the interview. For the patients not correctly taking their treatment and not correctly perform the instruction given, counseling was given immediately at the end of the interview. Confidentiality of the data was assured through omitting the participants’ name and assigning a code number.

### Data collection procedures and tools

Structured and validated tools were used to collect both the adherence status and explanatory variables, which were adopted from previous studies [22,24,34,35]. The tools were first prepared in English and translated into local language (Amharic) and back to English again to maintain its consistency.

Data collection was pursued in alignment with the study participant’s follow-up date. The data were collected by trained pharmacists and nurses through a face to face interview. Any misunderstanding during the interview process was amended through routine discussion with the principal investigator. Patients’ medical records were also reviewed to supplement further clinical data such as comorbidity, New York Heart Association (NYHA) functional class, hospitalization history.

#### Self-care behaviors data collection tool

Adherence to self-care recommendations regarding a low sodium diet, fluid restriction, regular exercise, weight monitoring, medication, and appointment keeping was measured using the “Revised Heart Failure Compliance Scale” [22,24,35] that has been successfully used to measure adherence in heart failure patients, demonstrating adequate reliability and validity [22,24,25,35,36]. This tool contains six questions with a five-point scale (always = 4, mostly = 3, half of the time = 2, seldom = 1, never = 0 points). Patients were asked to rate their adherence level in the past week (medication, low sodium diet, fluid restriction, and exercise), in the past month (daily/three times per week/weight monitoring), for the last 3 months (appointment keeping). Based on previous studies cutoff point used [22,35,36], for each individual self-care recommendation patients were classified as “Good adherent” when they followed a recommendation ‘always’ or ‘mostly’ and “Poor adherent” when they followed a recommendation half of the time, seldom or never. Finally, patients were considered to be **“**overall good adherent” when they adhered with ≥ 4 out of the 6 recommendations.

#### The tool used to assess HF patients’ knowledge

The patients’ HF knowledge was assessed as a factor for their adherence to self-care recommendations. The Japanese heart failure knowledge scale was used to assess the patients’ Knowledge [34]. This tool consists of questions which focuses on general HF knowledge, HF signs and symptoms, and HF related treatment and self-care. Its Cronbach’s alpha for internal consistency was 0.79.

Out of the 15 questions, 14 questions were used to assess patients HF knowledge with a choice of (yes, no, and I don’t know). Patients were asked one general question, five questions about HF signs and symptoms, and eight questions about HF self-care recommendations. The minimum and maximum possible score were 0 and 14, respectively. One point was given for each correct answer, no point was given for incorrect and ‘I don’t know’ responses. Based on previous similar studies [27,37] report, the overall level of knowledge was categorized as “Good” for HF patients who correctly answered ≥ 75% of knowledge questions and “Poor” for lower than 75%.

### Data processing and analysis

All the collected data were checked for completeness and consistency of responses manually. After cleaning, data were coded and entered into Epi Data version 3.1.

Then it was exported and analyzed using SPSS version 20. Both descriptive and analytical, statistical tests were utilized. Proportions and summary statistics were computed for the majority of independent variables. A binary logistic regression model was used to check the effect of different factors on patients’ adherence to self-care recommendations. In multivariable binary logistic regression analysis variables with the p-value<0.05 were considered statistically significant.

## Results

### Socio-demographic characteristics of study participants

A total of 310 heart failure patients participated in the study. The mean age of participants was 49 (± 19.5 SD) years with the range of 18 to 89 years. Out of 310 patients, 199 (64.2%) were women. Most of the participants 292 (94.2%) were Orthodox Christians. More than half 160 (51.6%) of the study participants were married and only 60 (19.4%) of patients were single. Almost half of the participants 153 (49.4%) were unable to read and write. Regarding occupation 119 (38.4%) of patients were housewife and only 20 (6.5%) of patients were government employed (Table 1).

**Table 1:** Socio-demographic and economic characteristics of heart failure patients, Gondar University referral hospital, Northwest Ethiopia, May 2017 (n = 310).

Variable		Frequency		Percentage	
**Age (in years)**					
<30		69		22.3	
30–49		70		22.6	
50–69		113		36.5	
≥70		58		18.7	
**Sex**					
Female		199		64.2	
Male		111		35.8	
**Religion**					
Orthodox		292		94.2	
Muslim		18		5.8	
**Marital status**					
Married		160		51.6	
Single		60		19.4	
Divorced		38		12.3	
Widowed		52		16.8	
**Palace of residence**					
Urban		170		54.8	
Rural		140		45.2	
**Educational level**					
Unable to read and write		153		49.4	
Able to read and write only		37		11.9	
Primary school (1–8)		61		19.7	
Secondary school (9–12)		37		11.9	
College /University		22		7.1	
**Occupation**					
Housewife		119		38.4	
Farmer		65		21.0	
Merchant		13		4.2	
Student		32		10.3	
Government employee		20		6.5	
Retired		13		4.2	
Others*		48		15.5	
Income (Ethiopian Birr/month)**					
<1000		127		41.0	
1000–2000		144		46.5	
2001–3000		21		6.8	
>3000		18		5.8	

Others* = daily labor and unemployment.

** 1USD = 28 Ethiopian Birr

### Clinical characteristics of study participants

Of 310 study participants, 138 (44.5%) of patients had chronic comorbidity. Of this hypertension (HTN) 79 (25.5%) and kidney disease (KD) 16 (5.2%) were the most common. The majority of the study participants was recently diagnosed as NYHA class III, 113 (36.5%) followed by class IV 88 (28.4%) heart failure. The participants’ median duration of diagnosis of heart failure was 36 months with a range of 2–360 months and majority 197 (63.5%) of patients had a hospitalization history (Table 2).

**Table 2:** Clinical profiles of heart failure patients, Gondar University referral hospital, Northwest Ethiopia, May 2017 (n = 310).

Variable	Frequency	Percentage
**Chronic comorbidity**		
None	172	55.5
HTN	79	25.5
KD	16	5.2
HTN+KD	12	3.9
Hyperthyroidism	16	5.2
DM	5	1.6
HIV	8	2.6
HTN + Hyperthyroidism	2	0.6
**NYHA functional class**		
I	42	13.5
II	67	21.6
III	113	36.5
IV	88	28.4
**Hospitalization history**		
Yes	197	63.5
No	113	36.5

DM-diabetes mellitus, HIV-human immunodeficiency virus, HTN-Hypertension, KD–Kidney disease

### Heart failure patients’ adherence to self-care recommendations

**Fig 1:**
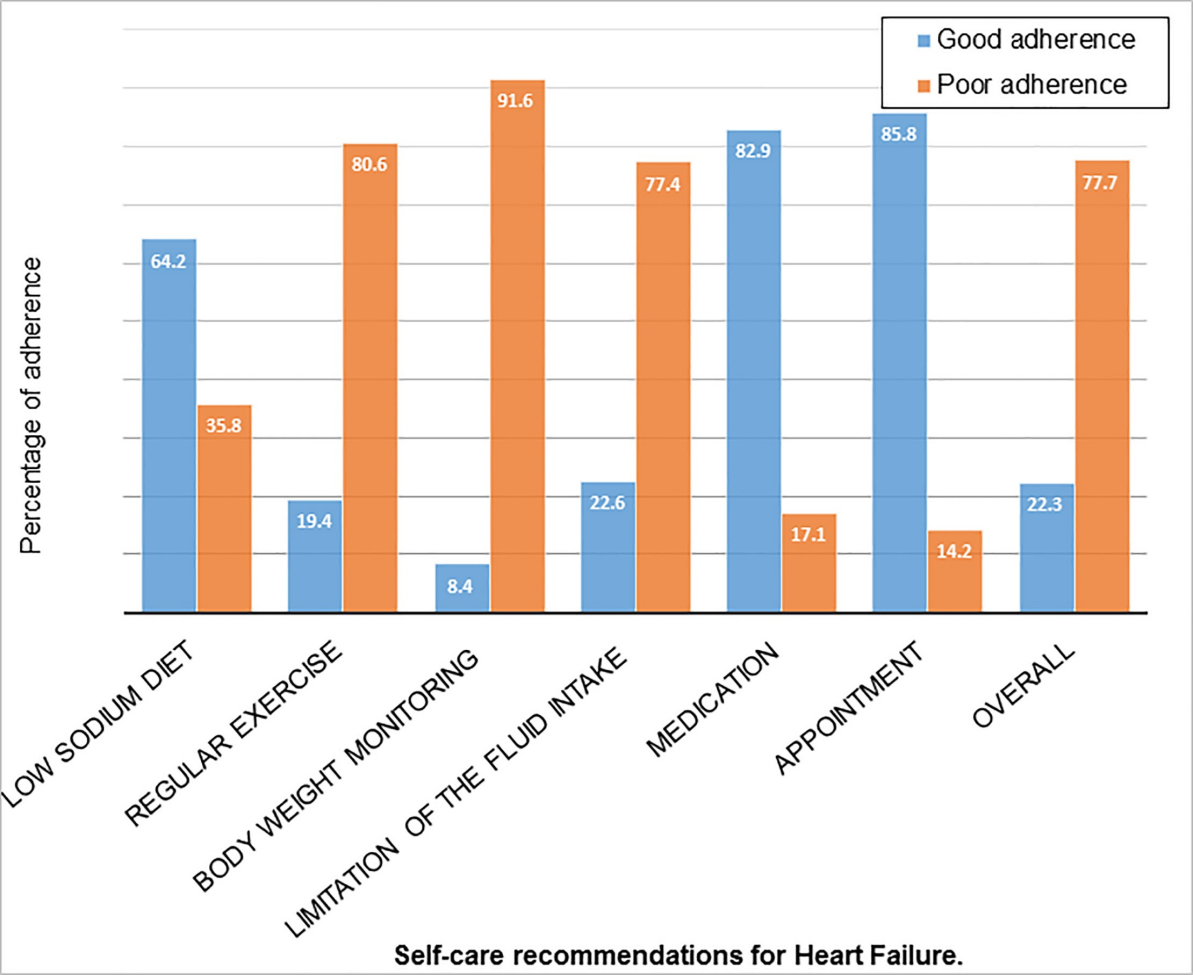
Adherence status of heart failure patients to self-care recommendations at Gondar University referral hospital, Northwest Ethiopia, May 2017 (n = 310).

Out of 310 study participants, only 69 (22%) had overall good adherence to their self-care recommendations with 95% CI (17.4%-26.8%). From individual self-care recommendation, higher levels of good adherence were noted for follow-up appointments 266 (85.8%) and taking prescribed medications as directed 257 (82.9%). However, most patients had higher levels of poor adherence to exercise 250 (80.6%), body weight monitoring 284 (91.6%), and fluid restriction 240 (77.4%) (Fig 1).

### Participants level of knowledge regarding heart failure

The mean (± standard deviation (SD)) HF knowledge score of participants was 8.4 ± 3 points with a range score of (0–14 points) out of a possible maximum score of 14. The majority of heart failure patients 232 (74.8%) had a poor level of knowledge regarding signs, symptoms and self-care management of heart failure.

### Factors associated with adherence to self-care recommendations

In the binary logistic regression analysis, the univariate analysis of adherence to self-care was positively associated with sex, educational level, comorbidity, and HF knowledge. After adjusting for all variables included in the univariate analysis; sex, comorbidity and HF knowledge remained significantly associated with good adherence to self-care recommendation in the multivariate analysis as depicted in Table 3. However, educational level was not a statistically significant factor (p = 0.257) associated with good adherence to self-care behaviors.

**Table 3:** Binary logistic regression analysis of factors associated with the overall adherence to self-care behaviors among heart failure patients at Gondar University referral hospital, Northwest Ethiopia, May 2017 (n = 310).

Variables	Overall adherence		COR With 95% CI	AOR with 95%CI	p-value
	Good	Poor			
	N = 69(%)	N = 241(%)			
**Age**					
<30	21(30.4)	48 (69.6)	2.38(0.991–5.722)	1.56 (0.419–5.821)	0.507
30–49	18(25.7)	52(74.3)	1.89(0.774–4.590)	1.77(0.583–5.364)	0.314
50–69	21(18.6)	92(81.4)	1.24(0.529–2.920)	1.40(0.535–3.635)	0.496
≥70	9(15.5)	49(84.5)	1	1	
**Gender**					
Male	34(30.6)	77(69.4)	2.07(1.201–3.565)**	**2.34(1.182–4.624)**	0.015*
Female	35(17.6)	164(82.4)	1	1	
**Marital status**					
Married	35(21.9)	125(78.1)	2.15 (0.847–5.439)	1.13 (0.394–3.245)	0.820
Single	20(33.3)	40(66.7)	3.83(1.402–10.482)**	0.67(0.146–3.323)	0.649
Divorced	8(21.1)	31(78.9)	2.04(0.645–6.483)	0.76(0.188–3.067)	0.699
Widowed	6(11.5)	46(88.5)	1	1	
**Place of residence**					
Urban	41(24.1)	129(75.9)	1.27(0.739–2.188)	1.28(0.631–2.615)	0.491
Rural	28(20.0)	112(80.0)	1	1	
**Educational level**					
No formal education	30(15.8)	160(84.2)	1	1	
Primary school	15(24.6)	46(75.4)	1.74(0.863–3.506)	1.40(0.609–3.240)	0.426
High school	16(43.2)	21(56.8)	**4.06(1.904–8.674)◆**	2.65(0.963–7.318)	0.059
College /University	8(36.4)	14(63.6)	3.05(1.176–7.897)*	1.14(0.278–5.142)	0.855
**Income**					
<1000	23(18.1)	104(81.9)	1	1	
1000–2000	34(23.6)	110(76.4)	1.40(0.772–2.529)	1.46(0.758–2.808)	0.259
2001–3000	5(23.8)	16(76.2)	1.41(0.470–4.250)	1.23(0.347–4.366)	0.748
>3000	7(38.9)	11(61.1)	2.88(1.007–8.221)*	2.90(0.700–12.04)	0.142
**Chronic comorbidity**					
None	49(28.5)	123 (71.5)	2.35(1.318–4.190)**	**2.57(1.280–5.142)**	0.008**
With comorbidity	20(14.5)	118(85.5)	1	1	
**NYHA class**					
I	9(21.4)	33(78,6)	1	1	
II	16(23.9)	51(76.1)	1.15(0.455–2.906)	1.28(0.464–3.524)	0.635
III	30(26.5)	83(73.5)	1.33(0.568–3.098)	1.33(0.531–3.329)	0.542
IV	14(15.9)	74(84.1)	0.69(0.273–1.763)	0.59(0.204–1.686)	0.322
**Knowledge level**					
Good	29(37.2)	49(62.8)	**2.84(1.604–5.032)◆**	**2.49(1.276–4.856)**	0.007**
Poor	40(17.2)	192(82.8)	1	1	

* *P*<0.05

**P<0.01

^**◆**^*P*<0.001

COR-Crude odds ratio, AOR- Adjusted odds ratio

NYHA- New York Heart Association

Accordingly, males were 2.34 (AOR = 2.34, 95% CI: 1.18–4.62) times more adherent than females. Regarding comorbidity, those who had no comorbid diseases were found to be 2.6 (AOR = 2.57, 95% CI: 1.28–5.14) times adherent than who had chronic comorbid conditions. Similarly, HF patients those who had a good level of knowledge were 2.5 times more adherent than patients who had a poor level of knowledge (AOR = 2.49, 95% CI: 1.28–4.86) (Table 3).

## Discussion

The results of the current study provide an insight to heart failure patients’ adherence to self-care recommendations. In this study, heart failure patients at Gondar University referral hospital had not been engaging in satisfactory self-care. Only 22.3% (95% CI; 17.4%-26.8%) of heart failure patients had good overall adherence to self-care recommendations. This is relatively comparable to the studies done in Sudan by Al-khader *et al* (28.95%) and in Ethiopia by Sewagegn *et al* (17.4%) of patients who had good overall adherence to self-care recommendations [22,27]. However, it is low compared to the study done in Atlanta by Marti *et al* (35.7%) and in the Netherlands by Van Der Wal *et al* (48%) of patients had been adherent to their self-care recommendations [25,30].

In this study, the level of adherence to body weight monitoring was (8.4%), regular exercise (19.4%), low sodium diet (64%), excess fluid intake restriction (23%), medication (83%) and appointment keeping (86%). In many other studies, the extent of adherence to each self-care recommendation was as follows: monitoring body weight (2.5%–83%) [22–26,30,31], doing regular exercise (21%–60%) [24, 26–28, 33, 36], follow a low sodium diet (28%–88%) [22–29], fluid restrictions (12%-90%) [22–27], taking prescribed medications as directed (75%–98.6%) [22,23,27,28], and more than 90% of heart failure patients’ keep their follow up appointments [26,27].

Although we realized that self-reported adherence might not completely reflect the actual practice, it is interesting to see that the adherence level of each individual self-care recommendation was almost comparable with other similar studies’ finding.

The disparities manifested on adherence to self-care recommendations among individual patient and between the studies might be due to the following rationale: First, the study population; for example, a study done by Lee *et al* only focuses on rural heart failure patients, whereas a study done by Ruf *et al* target on urban heart failure patients [23,26] while the current study focus on both rural and urban heart failure patients, this might affect the overall HF patients’ level of self-care practice. Second, the study design used, studies done by Nieuwenhuis *et al*, Van Der Wal *et al*, and Marti *et al* were a prospective follow-up study [24,25,30]. Thus, the long-term prospective follow-up study design used in these studies might provide accurate information for HF patients’ self-care practice unlike this cross-sectional study. Third, the geographical variation, while a couple of studies were done in developing countries [22,26,27], most of the studies were conducted in the developed nations. [23–25,28,30]. This might affect the living style and dietary habits of the study population and availability and accessibility of different aids which support the patients’ self-care behavior.

As described above this study only focus on the adherence sub-component of self-care behavior. It is better to understand that self-care behavior also includes HF patients health care provider consulting behavior while they experienced shortness of breath (SOB), a sign of legs/feet swelling, fatigue, and unusual weight gain[38,39].

The binary logistic regression analysis indicated that sex, comorbidity and patients’ knowledge regarding signs, symptoms and self-care management of heart failure were significantly associated with adherence to self-care recommendations. A paramount non-modifiable factor that has been associated with self-care adherence in this study was sex. Conflicting data exist regarding gender difference in heart failure patients’ self-care adherence. In the present study, males have exhibited 2.3 times more likely to be good adherent compared to female counterparts. Consistent findings were shown in studies done in South Africa and the Netherlands. Which revealed that men tended to be more adherent to their treatment than women [24,26]. In contrast, Ok *et al* reported that women had good adherence to self-care treatment than men [40]. Interestingly, many studies were divulged indifferent outcomes regarding gender difference and any relation with adherence to self-care [25,30,41–44]. A study has unveiled that women with HF and other cardiac conditions are more likely prone to psychosocial distress and need more social support than men [45]. Hence, the presence of psychological distress and lower social support were related to poor self-care in some studies report [28,36,46].

In this study, patients who had no chronic comorbid diseases were 2.6 times more likely to be good adherent than patients who had chronic comorbid conditions like HTN, KD, HIV, Hyperthyroidism, and Diabetes. This incongruity might be ascribed to a triple burden of polypharmacy, physical incapability and disease severity suffered by patients with comorbidity. Similar to this finding, Sewagegn *et al* and Kato *et al* reported that comorbidity significantly associated with adherence to self-care recommendations [25, 41]. This implicates that special attention and counseling should be given for heart failure patients with chronic comorbid conditions.

The other modifiable factor which significantly associated with adherence to self-care recommendation is the patients’ knowledge towards heart failure signs, symptoms, and self-care behavior. When compared, HF patients who had a good level of knowledge were 2.5 times more likely to be good adherent to their self–care recommendations than patients who had poor level of knowledge. It is anticipated that patients who were well acquainted about the signs, symptoms, and self-care behavior may perform more serious self-care than the patients who were unfamiliar. This finding is in line with studies done by Sewagegn et al, Ok *et al* and Matsuoka *et al*, as reported that lower HF knowledge significantly associated with heart failure patients poor adherence to self-care recommendations [27,37,40]. Improving patients' knowledge about HF is indispensable to improve self-care adherence and to decrease hospitalization, especially for rural HF patients [23,47]. Furthermore, some studies also reported that to improve heart failure patients’ adherence to self-care recommendations, better to focus on patient education to increase their knowledge. For these Shah D et al and Unverzagt S et al reported that the interdisciplinary team which consists of a nurse, pharmacist, registered dietician, social worker, physician, and a regular follow-up contacts of HF patients had an impact on their adherence to self-care behaviors[48–50].

In this study age, educational level, and NYHA functional class have not been strongly associated with adherence to self-care recommendations. However, several other studies reported different findings [23,24,27,30,42,51]. Sewagegn et al. reported that NYHA functional class was significantly associated with poor adherence to self-care treatment [27]. In contrast, the Marti *et al* study revealed that adherence was not associated with the functional capacity of the patient [30].

The current study has several limitations. Patients self-report during the interview was the primary source of data. Hence, recall bias and tendency to digress important information’s might affect the overall outcome of the study. Further, lower sample size and absence of multicenter data may hinder to generalize the study outcomes to the general population. Lastly, this is a cross-sectional study (had no follow-up evaluation) and this may affect the patients exact level of adherence to self-care behaviors and also difficult to conclude a causal relationship.

Bearing in mind these limitations this study finding may have an implication for healthcare professionals better to give more emphasis on counseling heart failure patients about HF signs, symptoms, and its management approaches to improve treatment adherence and overall prognosis.

## Conclusion

Heart failure patients’ adherence to self-care recommendation is frighteningly low, and selectively medication adherence and appointment keeping were good. Yet, adherence to low sodium diet, limiting excess fluid intake, doing a steady physical activity and weight monitoring was inadequate. Better adherence is associated with the absence of chronic comorbid diseases, being male in gender, and good level of HF knowledge. These results highlight a major opportunity for further prospective follow–up studies, which have an intervention approach for each self–care recommendations. "Although prior research found that self-reported adherence may be comparable to objectively measured adherence (e.g., from serum)[52], future studies assessing HF patient's adherence through objective measure are warranted to enhance our understanding in this topic."
